# Metal Oxide Nanolayer-Decorated Epitaxial Graphene: A Gas Sensor Study

**DOI:** 10.3390/nano10112168

**Published:** 2020-10-30

**Authors:** Marius Rodner, Adam Icardi, Margus Kodu, Raivo Jaaniso, Andreas Schütze, Jens Eriksson

**Affiliations:** 1 Applied Sensor Science Unit, IFM, Linköping University, 58183 Linköping, Sweden; marius.rodner@liu.se (M.R.); adaic903@student.liu.se (A.I.); 2 Institute of Physics, University of Tartu, 50411 Tartu, Estonia; margus.kodu@ut.ee (M.K.); raivo.jaaniso@ut.ee (R.J.); 3 Department of Systems Engineering, Lab for Measurement Technology, Saarland University, 66123 Saarbrücken, Germany; schuetze@lmt.uni-saarland.de

**Keywords:** metal oxide, epitaxial graphene on SiC, chemical gas sensor, nanolayer, inter-lab comparison

## Abstract

In this manuscript, we explore the sensor properties of epitaxially grown graphene on silicon carbide decorated with nanolayers of CuO, Fe_3_O_4_, V_2_O_5_, or ZrO_2_. The sensor devices were investigated in regard to their response towards NH_3_ as a typical reducing gas and CO, C_6_H_6_, CH_2_O, and NO_2_ as gases of interest for air quality monitoring. Moreover, the impact of operating temperature, relative humidity, and additional UV irradiation as changes in the sensing environment have been explored towards their impact on sensing properties. Finally, a cross-laboratory study is presented, supporting stable sensor responses, and the final data is merged into a simplified sensor array. This study shows that sensors can be tailored not only by using different materials but also by applying different working conditions, according to the requirements of certain applications. Lastly, a combination of several different sensors into a sensor array leads to a well-performing sensor system that, with further development, could be suitable for several applications where there is no solution on the market today.

## 1. Introduction

It is estimated that people in North America and Europe spend 90% of their time indoors, where the air quality is usually worse than in outdoor air. Poor indoor air quality is responsible for about 4 million premature deaths and costs about USD5 trillion in welfare costs per year [1,2]. One of the main challenges is to have a sensor solution that is accurate and reliable enough, while also being affordable [3]. Another big challenge is the miniaturization of such gas sensors, with reliable selectivity, stability, and sensitivity [4]. Moreover, IDTechEx expects that the total market for environmental sensors will be over USD3.8 billion by 2030 [5].

Two-dimensional materials such as graphene have been shown to exhibit outstanding sensitivity if used as gas sensors [6]. However, to achieve sensitivity and selectivity to desired target analytes, the surface normally needs to be functionalized. Often, metals or metal oxides are used for the functionalization, as there is a wide range of well-studied materials, which are not only well known in the scientific community but also dominate in commercial sensor applications [7]. A well-designed sensor focuses on a specific application, and the optimal material/gas combination already leads to very good sensor performance. To further increase the sensitivity, the surface-to-volume ratio of the sensing layers can be increased, as a larger detection area per unit volume results in more material/gas interaction due to a higher adsorption of gas molecules per volume. Especially when thinking about a miniaturization of the whole sensor device, nanostructured materials are often used with highly sensitive materials to combine their advantages. For example, the combination of sensitive and more selective nanostructured metal oxides with very sensitive and low noise graphene leads to promising sensor hybrids. There, the gas reaction mainly takes place on the metal oxide nanostructure, and the graphene is used as a highly sensitive transducer [8,9]. In this work, we show that decorating an epitaxially grown graphene surface with continuous metal oxide nanolayers (NLs) can lead to increased sensitivity and selectivity towards different desired target gases. The choice of material is based on previous observations. For example, we have shown that using Fe_3_O_4_ or TiO_2_ nanoparticles to decorate epitaxial graphene has very promising results regarding sensitivity [10,11]. However, controlled coverage of the whole surface with an NL is much easier compared to controlling the distribution of nanoparticles. Moreover, epitaxially grown graphene was found to outperform commercially available CVD graphene when used as a transducer in a gas sensor and decorated with V_2_O_5_ [12]. ZrO_2_ is used in many catalytic applications, such as the lambda sensor, and CuO was also found to be a very promising material for gas sensors [13,14]. Furthermore, the influence of several environmental properties on sensor performance is evaluated. The aim of this study is to provide an overview of how different material combinations affect sensor performance for a selection of typical toxic AQ pollutants.

## 2. Materials and Methods

The graphene surface was epitaxially grown on silicon carbide (SiC) through a silicon sublimation process [15]. As a semi-insulating 4H-SiC (0001) substrate is used, no further transfer of the grown graphene lattice is needed. Gold on titanium (Ti/Au, 2/200 nm) contact pads are sputtered onto the surface, and the resistance between the contacts is measured as a sensor response in the final device. More detailed information about the growth parameters, sensor fabrication, and the gas mixing system can be found in our previous works [10,12]. Gas measurements were performed with different gas mixing systems at Linköping University (LiU) [16] and Saarland University (UdS) [17,18]. Both laboratories use gas bottles with a purity of 6.0, and the gas mixing systems are calibrated on flow rather than concentration by using calibrated mass flow controllers (MFCs). To test the influence of UV irradiation on sensor response, two different UV LEDs, with wavelengths of 265 nm (Sensor Electronic Technology Inc, Colombia, GA, USA, S-T39B-F1-265-01-1-050) and 355 nm (Roithner Lasertechnik GmbH, Vienna, Austria, XSL-355-3E-R6), were used separately to illuminate the sensor layers.

The pulsed laser deposition (PLD) process used in this work is described in [12]. Copper oxide (CuO), iron (Fe) foil, vanadium pentoxide (V_2_O_5_), and zirconium dioxide (ZrO_2_) have been used as target materials. A krypton fluoride (KrF) excimer laser COMPexPro 205 (Coherent GmbH, Germany) at a wavelength of 248 nm and laser fluences between 3–7 J/cm^2^ have been used for ablation. The number of laser pulses was adjusted to achieve a layer thickness of around 0.5–1 nm. The sensor substrates were kept at room temperature during deposition.

In this manuscript, pristine epitaxial graphene on SiC will be referred to as PEG and decorated epitaxial graphene as DEG.

As mentioned before, the sensor resistance is measured over time, and the response is defined as

(1) $$Response=\frac{R_{gas}-R_{0}}{R_{0}}*100\%$$

where *R_gas_* is the saturated/absolute highest resistance signal during the gas exposure, and *R*_0_ corresponds to the baseline resistance before gas exposure.

## 3. Results

### 3.1. Morphological and Structural Characterization

**Figure 1:**
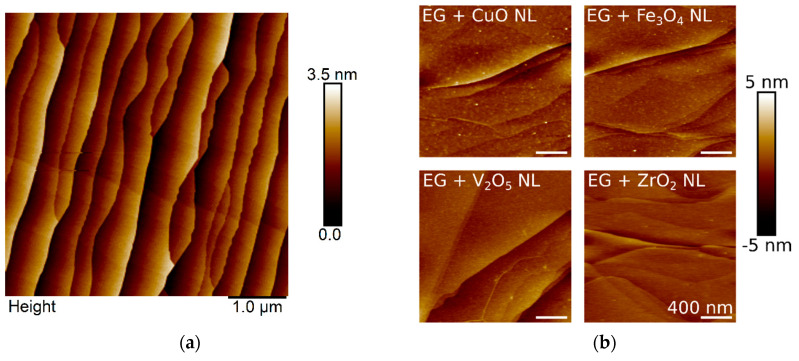
AFM images of pristine epitaxial graphene (PEG) (**a**) before and (**b**) after the decoration with nanolayers (NLs).

Figure 1a shows Atomic Force Microscopy (AFM) images obtained before the decoration of the graphene surface with the different metal oxide NLs, and Figure 1b shows a summary for all four surfaces after the decoration. The observed morphologies of the NL-decorated sensor surfaces are not very different compared to each other and still show a similar surface roughness compared to the pristine epitaxially grown graphene sample on SiC. The dominating features belong to the step-bunching edges formed at the SiC surface during high-temperature graphene growth. This is expected, as tapping mode AFM in ambient conditions does not allow the resolution of atomic structure, and the different layers form continuous films of a few atomic layers in thickness (0.5–1 nm) on top of a very flat substrate.

**Figure 2:**
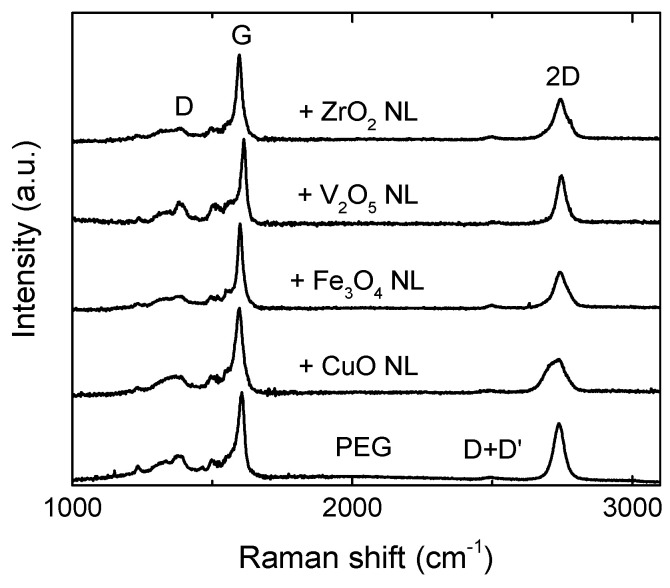
Raman spectra for PEG and after NL decoration.

To ensure that the integrity of the graphene lattice is still given after the surface decoration, Raman spectroscopy measurements have been performed (see Figure 2). For a better comparison, all spectra are normalized to the G peak. All spectra exhibit the typical G and 2D peaks around 1610 and 2737 cm^−1^, respectively [19]. The peak observed around 1350 cm^−1^ could be misinterpreted as the D peak, which is related to defects of the graphene lattice. Instead, these features, arising above 1280 cm^−1^ and extending into the G peak, are related to the interfacial buffer layer between the graphene and the SiC substrate [19]. The small full width at half maximum (FWHM) value of the 2D peak of the pristine graphene reference is an indication of a uniform monolayer of graphene [20]. After the surface decoration, the Raman spectrum stays approximately the same as the reference, indicating no induced structural damage. While the position of the 2D peak occurs at the same Raman shift for all samples, the G peak varies a bit for the different samples. It occurs at 1605 ± 10 cm^−1^. This could be due to different levels of doping in the graphene due to the charge transfer between the graphene and the various metal oxide decoration materials or simply due to different levels of strain in the graphene lattices [21]. It can also be seen that the 2D peaks broaden a bit after the surface decoration, and the ratios between G and 2D peaks slightly decrease. This could be related to some inclusions of bi- or multilayer graphene in the measured spot.

### 3.2. Gas Measurements

Ammonia (NH_3_) gas was used to generally study the response of the different hybrid sensors towards influences of operating temperature, relative humidity, and irradiation with UV light during the gas measurements. In addition, the sensor response to benzene (C_6_H_6_), formaldehyde (CH_2_O), nitrogen dioxide (NO_2_), and carbon monoxide (CO), i.e., gases of interest for, e.g., air quality monitoring, is discussed. The results were obtained over a period of about two years with the same sensors, showing that the sensors work without significant degradation during this time span if the operating temperature is kept at 150 °C or lower.

#### 3.2.1. Influence of Operating Temperature

It is well known that the operating temperature of a sensing layer has a huge impact on the sensor response, especially when using metal oxides or catalytic metals [22]. The sensor response was tested at several rather low temperatures, as the current sensor packaging limits the operating temperature to 200 °C. Therefore, no temperature above 150 °C was used.

**Figure 3:**
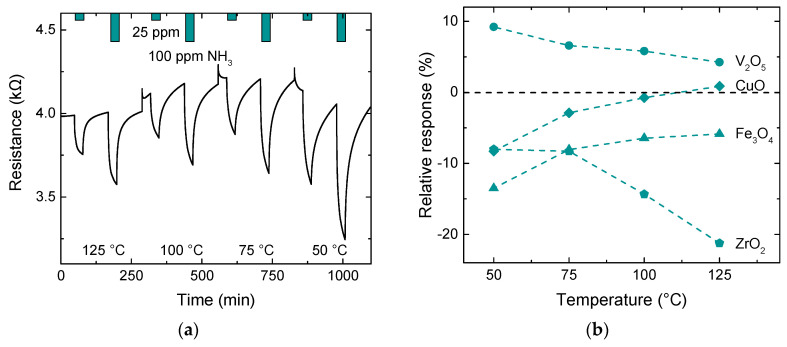
(**a**) Resistance over time of Fe_3_O_4_ NL decorated epitaxial graphene (DEG) vs. 25 and 100 ppm NH_3_ at different operating temperatures at 50% relative humidity (RH) and (**b**) relative responses of NL DEG samples for 25 ppm NH_3_ exposures at different operating temperatures at 50% RH.

All sensors have been tested at 125, 100, 75, and 50 °C at 50% relative humidity (RH) and exposed to 25 and 100 ppm NH_3_. Each exposure lasted for 30 min, with a relaxation phase of 90 min in between exposures, where the system was purged with synthetic air as background gas (80/20–N_2_/O_2_), also at 50% RH. The resistance over time is exemplarily shown for the Fe_3_O_4_ NL DEG sensor in Figure 3a, and the results for all sensors towards 25 ppm are shown in Figure 3b. The sharp peaks in resistance when changing temperatures are due to an overshoot in the temperature control mechanism. The highest observed absolute responses for each sample are highlighted in bold. It can be clearly seen that an increase in operating temperature does not always mean an increase in sensor response. Only ZrO_2_ has its highest response at 125 °C, while all other sensors exhibit their highest response at 50 °C. However, this rather untypical behavior with increasing temperature could be due to a reaction with OH groups or be products of reactions from OH groups and NH_3_ instead, leading to a higher overall response at the lower temperature, which is not necessarily related only to the NH_3_ exposure itself. This phenomenon was shown to occur for metal oxide gas sensors when operating them at relatively low temperatures in a humid environment [23,24]. While a higher sensor response is normally desired, the conditions at which this occurs may impose other disadvantages like higher time constants or a less stable sensor baseline. A perfect example of this behavior can be clearly seen in Figure 3a, as only exposures at 125 °C come close to a steady-state sensor response during the 30 min gas exposure. τ_63_ is extrapolated for all four operating temperatures using an exponential fit. It continuously increases, from approximately 150 s at 125 °C up to 550 s at 50 °C on average. Furthermore, the sensors are not always able to relax back to the baseline after 90 min when operated at lower temperatures. As a compromise between the level of response, response shape, and relaxation, all further measurements with ammonia were conducted at 75 °C except for ZrO_2_, which was operated at 125 °C. The overall highest response was observed for ZrO_2_ with a change of −21.2% for 25 ppm NH_3_ at 125 °C. Moreover, ZrO_2_ and Fe_3_O_4_ NL DEG sensors exhibit n-type behavior, i.e., resistance is reduced during exposure to reducing gases, while V_2_O_5_ exhibits p-type behavior. Presumably, this difference is due to different charge transfers between the metal oxide layer and the graphene, which will depend on the work function difference between the materials. CuO, on the other hand, shows a positive response at 125 °C and then switches to a negative response at lower temperatures. This change can also be induced at 125 °C when using a higher concentration (100 ppm) instead. The reason for this change in response direction is likely due to increasing gas adsorption, resulting in sufficient electrons being donated to the graphene for the Fermi level to move from below the Dirac point (p-type conductivity) to above the Dirac point (n-type conductivity). The change with temperature could also partly explain why the absolute response increases with lower temperatures as temperature and NH_3_ concentration work against each other in terms of charge carrier generation.

Other gases like benzene or formaldehyde, both volatile organic compounds (VOC), on the other hand, normally require a higher temperature to interact with the sensor surface. In a dry ambient, the four sensors were exposed to 200 ppb of C_6_H_6_ and CH_2_O at 50, 100, and 150 °C. Only CuO DEG was able to detect gas pulses down to 50 °C. While the ZrO_2_ DEG sensors showed a response only at 150 °C, Fe_3_O_4_ and V_2_O_5_ DEG sensors also exhibited a response at 100 °C.

#### 3.2.2. Influence of Relative Humidity

Besides the operating temperature, relative humidity in the ambient can be another critical parameter for sensor response. It was shown that many metal oxides exhibit a cross-sensitivity towards RH, and a higher level of RH in the ambient usually results in a lower gas sensitivity, as the target gas molecules compete with water molecules for available adsorption sites.

**Figure 4:**
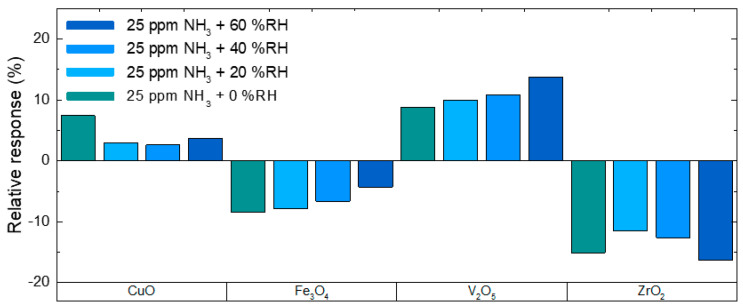
Relative responses of NL DEG samples for 25 ppm NH_3_ exposures at 75 °C (except ZrO_2_ at 125 °C) at different levels of RH.

Figure 4 summarizes the response magnitudes for all four NL DEG sensors towards 30 min exposures of 25 ppm NH_3_ at different levels of RH. The sensors were tested at approximately 0%, 20%, 40%, and 60% RH, and the operating temperature was kept at 125 °C (ZrO_2_) and 75 °C (all other NLs), respectively. Comparing the response at 0% to 20% RH, all sensors exhibit a decrease in response except V_2_O_5_, which not only increases for the first 20% RH but progressively increases from 8.8% at 0% RH to 13.8% at 60% RH. On the contrary, the response of the Fe_3_O_4_ NL DEG sensor decreases progressively with an increase in humidity, from initially −8.4% at 0% RH down to −4.3% at 60% RH. CuO, on the other hand, starts with a decrease in response for the first 20% RH but then stays almost constant for 40% and 60% RH. After the first drop in response at 20% RH compared to 0% RH, the ZrO_2_ NL DEG sensor increases its response for 40% and 60% RH again, eventually exceeding the response observed at 0% RH. This increase in response with an increase of relative humidity in the environment could be due to an additional sensor reaction with OH groups or with products resulting from reactions of OH groups with NH_3_, which can occur for metal oxide gas sensors when operated in a humid environment at relatively low temperatures [23,24]. An analysis of the change in baseline resistance due to the change in relative humidity can be found in the supplementary material (compare Table S1).

Table 1 summarizes several measurements of all sensors towards all measured gases when exposed in a dry (0% RH; <5 ppm H_2_O for gas from gas bottles (6.0) and <63.2 ppm for gas from a 0-air generator; see Section 3.3) or a humid (25% or 50% RH) environment. The measurements were performed in different laboratories, and the differences are discussed in Section 3.3. In this section, we show only one value per gas. All measurements were performed at 150 °C operating temperature, and the response was measured after 30 min of exposure. It can be clearly seen that the introduction of relative humidity can have a severe effect on the measured sensor response, e.g., no sensor was able to detect CO, C_6_H_6_, or CH_2_O in the presence of humidity. Similar to the observations for NH_3_, relative humidity enhances or hinders the response towards NO_2_. It greatly increases the response for V_2_O_5_ by almost 50% but only marginally increases it for ZrO_2_ by about 8% of the original response. The high response of the Fe_3_O_4_ NL DEG sensor decreases by about one-third. The response for CuO does not only decrease strongly, but it also changes direction from an increase in resistance towards NO_2_ exposure to a decrease in resistance if relative humidity is added. This is similar to what was observed for this sensor when exposed to NH_3_ + 50% RH at a temperature below 125 °C.

#### 3.2.3. Influence of UV Irradiation

The sensitivity of a chemical gas sensor was shown to be enhanced through UV irradiation for many different material/gas combinations, and, normally, the irradiation also decreases time constants, hence speeding up the sensor response [25]. Moreover, it was shown that UV irradiation can be used to clean graphene surfaces, thus freeing active sites for a target gas interaction [26]. Through the irradiation with light, photons can react with the target gas or the sensing material, which depends on the possibility of adsorbing photons at the given photon energy. Therefore, certain wavelengths work best for a given material/gas combination, and longer wavelengths will not have an effect at all.

**Figure 5:**
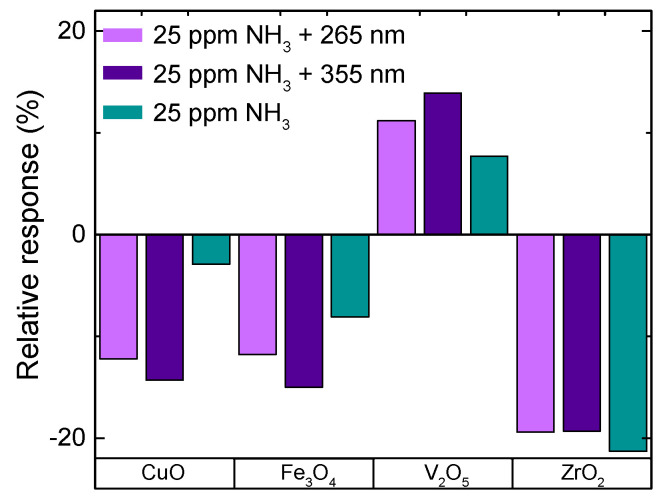
Relative responses of NL DEG sensors vs. 25 ppm of NH_3_ at 50% RH, without and with UV irradiation, based on [27].

Figure 5 summarizes the responses towards a 30 min exposure of 25 ppm NH_3_ at 50% RH without and with the influence of UV irradiation. The sensors were operated at 75 °C except for ZrO_2_, which was operated at 125 °C. A clear trend can be observed as the relative sensor response is highest with 355 nm UV irradiation and decreases with no irradiation for all sensors except ZrO_2_. For ZrO_2_, on the other hand, a small decrease is observed with UV irradiation (−19.3%) compared to −21.3% without irradiation. When changing the UV irradiation from 355 to 265 nm, the sensor response decreases slightly. Both wavelengths have a strong impact on the time constants, which are lower with additional UV light compared to no irradiation. With UV irradiation, the time constants range between 350–500 s, whereas without irradiation, they go up to 550–1000 s. In contrast to the small effect on the response when adding UV irradiation, the ZrO_2_ DEG sensor shows the highest enhancement in time constant from 1000 s down to 450 s.

The complete sensing mechanism of DEG is not fully understood yet, and the introduction of one more variable like UV irradiation results in additional complexity. It is known that if the bandgap is below the photon energy (e.g., 3.49 eV for 355 nm), there will be charge excitation, changing the charge density in the surface modification and graphene as well [25]. This effect should be more distinct for thicker surface layers. This is in agreement with the observation that only ZrO_2_, with its comparably large bandgap (>5 eV), shows no significant difference in response magnitude under UV irradiation, while all other sensors did exhibit a significant difference (CuO ≈ 1.4 eV, Fe_3_O_4_ ≈ 3 eV, and V_2_O_5_ ≈ 2.4 eV).

### 3.3. Interlab Studies

To verify that measurements performed are valid regardless of the lab-setting, some measurements were first performed in the gas sensing lab at Linköping University (LiU) and then repeated in another lab at Saarland University (UdS). All measurements were performed at 150 °C operating temperature, but the tested gas concentrations and levels of relative humidity were not always the same due to some restrictions of the respective systems. For example, the level of humidity and the gas concentrations changed between 25% RH and 200 ppb for LiU and 50% RH and 500 ppb for UdS, respectively. This means that a higher sensor response is expected for measurements performed at UdS as a higher concentration was used. Nevertheless, we tried to keep everything as close as possible to support the comparison. Measurements that did not show a response at both labs are not included (e.g., CH_2_O with humidity; compare Section 3.2.2). An overview of the comparison is given in Table 2. If exposed to C_6_H_6_ and CH_2_O, the response measured at UdS is lower for all sensors except for V_2_O_5_. Especially for the CuO DEG sensor, the response varies a lot. The reported response for ZrO_2_ towards C_6_H_6_ was only observed at LiU, but the response (0.02%) was very small. Therefore, the measurements conducted with VOCs do not result in a clear outcome for the interlab studies. On the other hand, measurements with exposures towards NO_2_ show the same trend for three of the four sensors: the response increases with an increase in concentration and decreases with the introduction of relative humidity, indicating that the sensors show very similar performance at both laboratories. The only exception is the exposure of the CuO NL towards 200 ppb at 25% RH at LiU, where the response direction changed compared to the exposure at 0% RH or towards 500 ppb at 50% RH at UdS, which could be explained by the competing effects of NO_2_ and RH on the sensor surface.

The single standard deviation of the baseline resistance was used as an indicator of noise. Since a similar electronic setup was used in both labs, there is no distinct trend in the noise level between the labs. The standard deviation stayed below 0.1 Ω for all sensors, leading to a worst-case relative noise level of approximately 0.007%. This highly depends on the baseline resistance of the different sensors, which varied between 1.5 to 3.2 kΩ. With the ZrO_2_ DEG sensor, the relative baseline noise was even 10 times lower.

Additional deviations could come from the uncertainties in concentration from the gas bottles, as each bottle, even with a purity of 6.0, has an approximate error range in concentration of up to ±10%. This alone can have a major impact on the observed sensor response. Another problem could come from the administered flows from the MFCs, although this is rather unlikely as only calibrated MFCs are used and frequently reassessed. Moreover, the background gas (purity of 6.0) used for mixing of test gases still contains approximately 1 ppm of contaminants, or the system could be contaminated by prior gas tests [28]. This can especially influence the comparison between both labs as the background gas at LiU is mixed from gas bottles (N_2_ and O_2_), while the background gas at UdS comes from a 0-air generator. Since only relative responses and not absolute values are compared, the comparison is still valid. A variation due to the very small difference in background humidity is not expected (compare Section 3.2.2).

Nevertheless, we think that interlab studies should be conducted to verify sensor performances, at least qualitatively, and to raise awareness that published results may only be accurate for the specific setup used for the investigation, which itself can have an influence on sensor response. Indepth studies, comparing their gas measurements with certified labs, have shown that even there, unexpected deviations can occur, which opens the possibility for more insight into sensor behavior [29,30]. Unfortunately, round-robin testing, which is the standard in analytical chemistry to ensure that experimental procedures are correct, is still not applied in the field of chemical sensor research [31,32].

### 3.4. Data Analysis/Multisensor Array

As we have seen in the earlier sections, the different NL DEG sensors respond differently to different gases and environmental influences. This is summarized in Table 3. If the gas has the addition “+RH”, either 25% or 50% RH was used, which did not matter for the direction of response in this case. The addition “+UV” means that the measurement was performed with 355 nm UV LED and at 50% RH.

**Figure 6:**
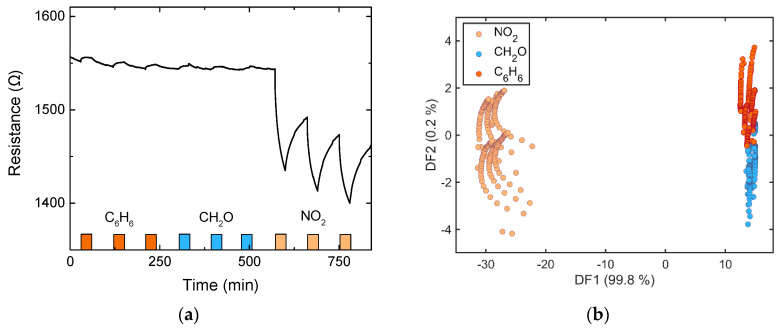
(**a**) Resistance over time of V_2_O_5_ NL DEG towards 200 ppb of C_6_H_6_, CH_2_O, and NO_2_ at 0% RH and 150 °C and (**b**) linear discriminant analysis (LDA) based on gas exposures of all four sensors.

However, one sensor alone would not be able to distinguish between different gases very effectively. Therefore, using a sensor array with all or some of the used sensors could lead to a better classification of exposed gases [33]. A simple LDA (linear discriminant analysis) [34] was used to differentiate between three gases (C_6_H_6_, CH_2_O, and NO_2_) applied to all sensors during several single gas exposures at 0% RH. Each gas was applied three times, with a concentration of 200 ppb and a sensor operating temperature of 150 °C. The gas cycle was repeated twice, resulting in six exposures per gas in total. DAV^3^E, a MATLAB toolbox [35], was used to calculate an LDA scatter plot based on each measurement point during the exposure of each sensor. Each measurement point is used as this is exactly what would happen if the sensors are used in a real setup, where the resistance over time is measured and gas exposures need to be determined. For better clarity, however, only every 50th point is plotted. The first five seconds of each exposure are neglected due to time constants of the gas mixing system. The result of this LDA is shown in Figure 6. As expected, it is rather easy to discriminate between NO_2_ and the two VOCs, as the response towards NO_2_ is much higher in comparison. It is harder, but still not impossible, to discriminate between benzene and formaldehyde. This example is very much oversimplified as, for example, similar concentrations of different gases might give very different magnitudes of response (e.g., NO_2_ vs. VOCs). Another problem is the high time constants of the sensor, and, therefore, each measurement point was used and not the slope of the change of the signal during exposure. One way to overcome this issue would be the application of cycled methods like temperature cycled operation (TCO) to create steep changes and more transient data for evaluation [33,36,37]. A more extensive data set with different gases, concentration levels, and combined gas exposures would be needed to make a proper statement about the applicability of these exact four sensors to real-world applications, which, in turn, would be highly application-dependent.

## 4. Conclusions

In conclusion, we have shown that nanolayer-decorated epitaxial graphene sensors perform differently depending on the decoration material used and the measurement conditions. Increased operating temperature and level of relative humidity were found to either increase or decrease the sensor response towards NH_3_, depending on the material. Moreover, illumination with UV light, in general, results in increased sensor response and decreased time constants, although some decoration materials are not significantly affected by UV. Investigating gases of interest for AQM, the influence of relative humidity is large as it inhibits the response towards C_6_H_6_, CH_2_O, and CO completely, at least at the low concentrations tested here. Comparing the sensors in an interlab study, the responses vary strongly for the measured VOCs but stay very comparable for NO_2_. This could indicate significant variations in either the concentrations of the source gases or, more likely, the concentrations of impurities in the source gas bottles, which, in turn, is an important aspect to consider for sensor calibration. Combining all four sensors into a sensor array, it was possible to distinguish between C_6_H_6_, CH_2_O, and NO_2_ in a dry ambient using an LDA.

## Figures and Tables

**Table 1:** Summary of relative responses towards CO, C_6_H_6_, CH_2_O, and NO_2_ in dry and humid conditions.

	Relative Response (%)							
	CO 500 ppb 0% RH	CO 500 ppb 50% RH	C_6_H_6_ 200 ppb 0% RH	C_6_H_6_ 200 ppb 25% RH	CH_2_O 200 ppb 0% RH	CH_2_O 200 ppb 25% RH	NO_2_ 200 ppb 0% RH	NO_2_ 200 ppb 25% RH
**CuO**	0.12	/	2.81	/	1.54	/	5.28	−0.89
**Fe_3_O_4_**	0.09	/	0.40	/	0.33	/	39.69	26.68
**V_2_O_5_**	0.14	/	0.17	/	0.25	/	−5.84	−8.65
**ZrO_2_**	/	/	0.02	/	/	/	−14.60	−15.75

**Table 2:** Summary of responses of all sensors towards C_6_H_6_, CH_2_O, and NO_2_ at 150 °C under dry and humid conditions.

	Relative Response (%)							
	C_6_H_6_ 200 ppb 0% RH LiU	C_6_H_6_ 500 ppb 0% RH UdS	CH_2_O 200 ppb 0% RH LiU	CH_2_O 500 ppb 0% RH UdS	NO_2_ 200 ppb 0% RH LiU	NO_2_ 500 ppb 0% RH UdS	NO_2_ 200 ppb 25% RH LiU	NO_2_ 500 ppb 50% RH UdS
**CuO**	2.81	0.45	1.54	0.92	5.28	14.60	−0.89	5.87
**Fe_3_O_4_**	0.40	0.20	0.33	0.19	39.69	65.82	26.68	60.63
**V_2_O_5_**	0.17	0.27	0.25	0.38	−5.84	−8.93	−8.65	−11.87
**ZrO_2_**	0.02	/	/	/	−14.60	−17.10	−15.75	−16.94

**Table 3:** Summary of response directions (+ for positive, − for negative, and/for no response) of all sensors towards CO, C_6_H_6_, CH_2_O, NH_3_, and NO_2_ under dry and humid conditions.

	Qualitative Response										
	CO	CO + RH	C_6_H_6_	C_6_H_6_ + RH	CH_2_O	CH_2_O + RH	NH_3_	NH_3_ + RH	NH_3_ + UV	NO_2_	NO_2_ + RH
**CuO**	+	/	+	/	+	/	+	+	−	+	−
**Fe_3_O_4_**	+	/	+	/	+	/	−	−	−	+	+
**V_2_O_5_**	+	/	+	/	+	/	+	+	+	−	−
**ZrO_2_**	/	/	+	/	/	/	−	−	−	−	−

## Supplementary Materials

The following are available online at https://www.mdpi.com/2079-4991/10/11/2168/s1, Table S1: Summary of changes of baseline resistance with varying relative humidity.
