# Supplementary material for: Metal Oxide Nanolayer-Decorated Epitaxial Graphene: A Gas Sensor Study
Source: Nanomaterials (Basel). 2020 Oct 30;10(11):2168. doi: 10.3390/nano10112168 (PMC7716239; doi:10.3390/nano10112168)
Supplement: Supplementary file 1 [file nanomaterials-10-02168-s001.docx]

Supplementary Material

Metal Oxide Nanolayer-Decorated Epitaxial Graphene: A Gas Sensor Study

Marius Rodner ^1^, Adam Icardi ^1^, Margus Kodu ^2^, Raivo Jaaniso ^2^, Andreas Schütze ^3^ and Jens Eriksson ^1,^*

^1^ Applied Sensor Science Unit, IFM, Linköping University, 58183 Linköping, Sweden; marius.rodner@liu.se (M.R.); adaic903@student.liu.se (A.I.)

^2^ Institute of Physics, University of Tartu, 50411 Tartu, Estonia; margus.kodu@ut.ee (M.K.), raivo.jaaniso@ut.ee (R.J.)

^3^ Department of Systems Engineering, Lab for Measurement Technology, Saarland University, 66123 Saarbrücken, Germany; schuetze@lmt.uni-saarland.de

***** Correspondence: jens.eriksson@liu.se; Tel.: +46-(0)-13-286-613

Table S1 summarizes the changes of baseline resistance for all four sensors depending on a change in relative humidity in the ambient. It can be clearly seen that a change from any level of relative humidity to one that is not zero, the change in baseline resistance is rather small and sometimes not even observable (e.g. V_2_O_5_ and ZrO_2_ when changing from 60 to 40% RH). A change from 20 to 0% RH^[[1]](#footnote-1)^ on the other hand, has a rather large impact on the baseline resistance, which changes for about 3–4.5%. Only the ZrO_2_ decorated EG sensor shows almost no change in baseline resistance, but this is most probably due to that it is operated at 125 °C and not at 75 °C as all the other sensors. In general, a reduction in relative humidity can be seen as some kind of n-type doping, as water itself is a p-dopant and an introduction of more water would therefore be p-doping. Accordingly, CuO and V_2_O_5_ decorated EG (as p-type sensors) experience a decrease in resistance when decreasing the relative humidity, and Fe_3_O_4_ and ZrO_2_ decorated EG (as n-type sensors) react inversely. This is in agreement with the findings of measurements with NH_3_ as a reducing gas where the sensor responses are exactly opposite of what happens when decreasing the relative humidity.

**Table S1.** Summary of changes of baseline resistance when varying relative humidity in the ambient at 75 °C (except ZrO_2_ at 125 °C).

|  | **Baseline change** | | |
| --- | --- | --- | --- |
|  | **Relative humidity (%)** | **Absolute change (Ω)** | **Relative change (%)** |
| **CuO** | 60 to 40 | −2.6 | −0.06 |
|  | 40 to 20 | −7.8 | −0.18 |
|  | 20 to 0 | −197.2 | −4.54 |
| **Fe_3_O_4_** | 60 to 40 | 6.5 | 0.12 |
|  | 40 to 20 | 18.1 | 0.34 |
|  | 20 to 0 | 228.5 | 4.26 |
| **V_2_O_5_** | 60 to 40 | / | / |
|  | 40 to 20 | −66.4 | −0.58 |
|  | 20 to 0 | −365.0 | −3.18 |
| **ZrO_2_** | 60 to 40 | / | / |
|  | 40 to 20 | 2.1 | 0.10 |
|  | 20 to 0 | 1.0 | 0.05 |

1. < 5 ppm H_2_O for gas from gas bottles (6.0) and < 63.2 ppm for gas from a 0-air generator (see Section 3.3) [↑](#footnote-ref-1)
